# Prevalence and characteristics of metabolic syndrome and its components among adults living with and without HIV in Nigeria: a single-center study

**DOI:** 10.1186/s12902-023-01419-x

**Published:** 2023-07-28

**Authors:** Jibreel Jumare, Patrick Dakum, Nadia Sam-Agudu, Peter Memiah, Rebecca Nowak, Florence Bada, Uzoamaka Oguama, George Odonye, Ruxton Adebiyi, Cristiana Cairo, Vivian Kwaghe, Clement Adebamowo, Alash’le Abimiku, Man Charurat

**Affiliations:** 1 Institute of Human Virology, University of Maryland School of Medicine, Baltimore, MD 21201 USA; 2 International Research Center of Excellence, Institute of Human Virology Nigeria, Federal Capital Territory, Abuja, Nigeria; 3 University of Abuja Teaching Hospital, Abuja, Nigeria; 4 Department of Epidemiology and Public Health, and Greenebaum Comprehensive Cancer Center, University of Maryland School of Medicine, Baltimore, MD 21201 USA

**Keywords:** Metabolic syndrome disorders HIV Nigeria prevalence characteristics

## Abstract

**Background:**

Persons living with HIV (PLHIV) now live longer due to effective combination antiretroviral therapy. However, emerging evidence indicates that they may be at increased risk for some cardiometabolic disorders. We compared the prevalence of metabolic syndrome (MetS) and its component disorders between persons living with and without HIV in Nigeria.

**Methods:**

This was a cross-sectional analysis of baseline data from a prospective cohort study of non-communicable diseases among PLHIV along with age- and sex-matched persons without HIV (PWoH) at the University of Abuja Teaching Hospital Nigeria. We collected sociodemographic and clinical data, including anthropometric measures and results of relevant laboratory tests. MetS was defined using a modification of the third report of the National Cholesterol Education Program Adult Treatment Panel (NCEP ATP III) criteria.

**Results:**

Of the 440 PLHIV and 232 PWoH, women constituted 50.5% and 51.3% respectively. The median age of the PLHIV was 45 years while that of the PWoH was 40 years. The prevalence of MetS was 30.7% (95% CI: 26.4%, 35.2%) and 22.8% (95% CI: 17.6%, 28.8%) among the PLHIV and PWoH respectively (*P* = 0.026). Independent associations were found for older age (*P* < 0.001), female sex (*P* < 0.001), family history of diabetes (*P* < 0.001), family history of hypertension (*P* = 0.013) and alcohol use (*P* = 0.015). The prevalence of component disorders for PLHIV versus PWoH were as follows: high blood pressure (22.3% vs 20.3%), prediabetes (33.8% vs 21.1%), diabetes (20.5% vs 8.2%), high triglycerides (24.5% vs 17.2%), low HDL-Cholesterol (51.1% vs 41.4%), and abdominal obesity (38.4% vs 37.1%). Adjusting for age and sex, prediabetes, diabetes, and low HDL-Cholesterol were significantly associated with HIV status. Duration on antiretroviral therapy, protease inhibitor-based regimen, CD4 count, and viral load were associated with some of the disorders mostly in unadjusted analyses.

**Conclusion:**

We found a high burden of MetS and its component disorders, with significantly higher prevalence of dysglycemia and dyslipidemia among PLHIV as compared to PWoH. Integration of strategies for the prevention and management of MetS disorders is needed in HIV treatment settings.

**Supplementary Information:**

The online version contains supplementary material available at 10.1186/s12902-023-01419-x.

## Background

The advent of combination antiretroviral therapy (ART) has dramatically improved life expectancy among persons living with the human immunodeficiency virus (PLHIV) [1]. With a restored prospect of longevity, which now approaches that of persons without HIV (PWoH), PLHIV experience similar background risks for cardiometabolic disorders and other non-communicable diseases (NCDs) as the general population [2]. Beyond this, there are indications that they may be at an even higher risk for some of these disorders [3, 4]. Multiple hypotheses have been postulated to explain this, including accelerated aging, chronic immune activation, immune-senescence and adverse effects of antiretroviral medications [5].

The pathophysiology and treatment of HIV have been linked to metabolic syndrome (MetS), a cluster of interrelated disorders comprising high blood pressure, dyslipidemia, dysglycemia and abdominal obesity [6, 7]. This constellation of metabolic disorders has been shown to be a strong predictor of future cardiovascular events [8, 9], and therefore has significant public health implications among PLHIV as in the general population. The reported prevalence of metabolic syndrome has varied widely (6.2%-58%) [10, 11]. Observed differences in estimates could be attributed to definitional criteria used, characteristics of study participants and treatment era during which these studies were conducted.

ART regimens have evolved over time, and some agents with profound metabolic toxicity have been withdrawn from the treatment armamentarium [12]. Conversely, recent developments in ART approaches may have inadvertently introduced other sources of adverse metabolic effects. Notable among these is the recommendation for low- and middle-income countries to transition from non-nucleoside reverse transcriptase inhibitors (NNRTIs) to dolutegravir (DTG), an integrase strand transfer inhibitor (INSTI), which may be associated with a worse metabolic outcome despite its overall favorable profile [13]. Moreover, the widespread implementation of WHO-recommended universal ‘test and start’ treatment strategy [14] might prolong the duration of exposure to antiretroviral drugs and potentially lead to earlier onset of metabolic adverse events. The cumulative impact of these changes on the burden of metabolic syndrome disorders among PLHIV needs to be further explored.

In this report, we examined characteristics associated with MetS and its components, in addition to presenting estimates of prevalence, among PLHIV along with age- and sex-matched PWoH in a large NCD evaluation cohort in Nigeria.

## Methods

### Study design and setting

This was a cross-sectional analysis of baseline data obtained from participants in a prospective cohort study of NCDs among PLHIV and PWoH underway at the University of Abuja Teaching Hospital (UATH) Nigeria, an HIV treatment facility supported by the Institute of Human Virology Nigeria’s United States President’s Emergency Plan for AIDS Relief (PEPFAR) program.

### Study participants

Prospective participants were screened based on the following eligibility criteria. Inclusion: aged 18 years or older; provided informed consent; documented evidence of HIV status; and willing to undergo study procedures. Exclusion: major medical or psychiatric condition that would interfere with study activities. Following ethical approval, enrollment of participants began in February 2021 at the HIV care and treatment facility as well as the counseling and testing center of UATH. PLHIV attending clinical follow up and persons testing negative to HIV were consecutively recruited. Data for 440 PLHIV and 232 PWoH were included in this analysis. The PWoH were frequency-matched to the PLHIV by age and sex using age ≤ 40 and > 40 years to achieve balance in proportions. Informed consent was obtained from all participants and study procedures were approved by Institutional Review Boards of the University of Maryland Baltimore (HP-00093307), University of Abuja Teaching Hospital (UATH/HREC/PR/2020/005/10), and the Federal Capital Territory Abuja (FHREC/2020/01/37/04–05-20).

### Clinical assessment

Research nurses used standardized questionnaires to document general medical information, including medication and comorbidity history, and measurements of blood pressure (BP), weight, height, as well as waist and hip circumference [15, 16]. Relevant components of the WHO STEPS instrument were adopted [17]. Weight and height were measured using ‘Health o Meter 500KL Eye Level Digital Medical Scale’, blood pressure was measured with ‘Omron Automatic Professional Digital Blood Pressure Monitor (HEM-907XL)’, while waist and hip circumference were measured using a flexible non-elastic tape measure following standard recommendations. Hypertension was defined as systolic BP ≥ 140 mmHg or diastolic BP ≥ 90 mmHg (in line with the cut-off of the 6^th^ report of the Joint National Committee [JNC VI]) [18] or history of treatment for hypertension. Weight and height were used to determine body mass index (BMI), calculated as the ratio of weight [in kilograms (Kg)] to the square of height [in meters squared (m^2^)]. Overweight and obesity were defined by BMI 25- < 30 kg/m^2^ and BMI ≥ 30 kg/m^2^ respectively [19]. Abdominal obesity was defined as waist-to-hip ratio ≥ 0.85 or waist girth ≥ 80 cm for females and waist-to-hip ratio ≥ 0.9 or waist girth ≥ 94 cm for males [11]. Diabetes mellitus was defined as fasting blood sugar (FBS) ≥ 126 mg/dl or random blood sugar (RBS) ≥ 200 mg/dl or glycosylated hemoglobin (HbA1c) ≥ 6.5% or history of treatment for diabetes. Prediabetes was defined as FBS 110–125 mg/dl or RBS 140–199 mg/dl or HbA1c 5.7–6.4%. Dyslipidemia was assessed using serum triglyceride and high-density lipoprotein cholesterol (HDL-C) levels. Hypertriglyceridemia was defined as serum triglyceride level ≥ 1.7 mmol/L, while low HDL-C was defined as serum HDL-C ≤ 1.04 mmol/L for males and ≤ 1.3 mmol/L for females [11]. We used a modification of the National Cholesterol Education Program’s (NCEP) Adult Treatment Panel (ATP III) criteria to define metabolic syndrome. Participants with ≥ 3 of the following fulfilled the definition for MetS: HbA1c ≥ 5.7% or RBS ≥ 140 mg/dl (used in place of FBS ≥ 100 mg/dl); hypertriglyceridemia as defined above; low HDL-C as defined above; abdominal obesity as defined by waist circumference specified above; and systolic BP ≥ 130 mmHg or diastolic BP ≥ 85 mmHg.

### Laboratory assessment

Participants’ blood samples were analyzed to determine HIV-1 serological status and for the measurement of viral load (limit of detection: 20 copies/mL) and CD4 + T-cell count [20]. A ‘Vitros® 350’ chemistry analyzer was utilized for metabolic panel assays, including glucose and lipids. HbA1c level was measured using Finecare™ rapid quantitative analyzer that employs fluorescent immunoassay methods. These were performed at the Institute of Human Virology Nigeria-supported Training Laboratory located in Asokoro, Abuja.

### Statistical analysis

Demographic and clinical characteristics were compared between PLHIV and PWoH using Pearson chi-square or Fisher’s exact test for categorical variables and t-test or Wilcoxon test for continuous variables. Logistic regression analyses were done to assess the odds of each metabolic disorder, comparing the PLHIV to PWoH, adjusting for age and sex. Sociodemographic and clinical characteristics were explored for independent associations with individual metabolic disorders in multivariable logistic regression models among all participants, and for HIV-related factors, among the PLHIV. Variables with *p*-value < 0.1 in univariable analysis were included in the multivariable models. All statistical analyses were performed using SAS version 9.4 software (SAS Institute, Cary, North Carolina).

## Results

### Demographic and clinical characteristics

The median age of participants was 42 years. The PLHIV were slightly older than the PWoH (Median age: 45 and 40 years respectively; *P* < 0.001) (Table 1). Sex distribution was balanced, with women constituting approximately 50% of the cohort, and this was similar among PLHIV and PWoH (*P* = 0.686). One in five participants only completed primary/elementary level education or less, and the PLHIV were more likely to have lower levels of education compared to the PWoH (*P* < 0.001). Overall, the PLHIV were more likely to be employed (*P* < 0.001) and had significantly higher income (*P* = 0.002). While there was a relatively higher proportion of married individuals among the PLHIV, they were more likely to be widowed, divorced, or separated (*P* < 0.001). The vast majority of this cohort never smoked or used alcohol (93.6% and 86.6% respectively) but the PLHIV had significantly higher percentage of ever users of alcohol (*P* = 0.017). There was no significant difference in level of leisure-time physical activity comparing PLWH (13.4%) with PWoH (9.6%) (*P* = 0.151) (Table 1).

**Table 1:** Demographic and clinical characteristics

Characteristics	N	All 672	PLHIV 440	PWoH 232	*P*-value
**Age (years), Median (Q1, Q3)**	672	42 (34, 50)	45 (36, 52)	40 (26, 46.5)	< 0.001^W^
**Gender, Female n (%)**	672	335 (49.9)	222 (50.5)	119 (51.3)	0.686^F^
**Education, n (%)**	672				< 0.001^F^
** None/Primary**		139 (20.7)	105 (23.9)	34 (14.6)	
** Junior/Senior Secondary**		236 (35.1)	163 (37.0)	73 (31.5)	
** Tertiary**		297 (44.2)	172 (39.1)	125 (53.9)	
**Occupation, n (%)**	672				< 0.001^F^
** Civil Service/Governmental**		152 (22.6)	100 (22.7)	52 (22.4)	
** Non-governmental**		68 (10.1)	21 (4.8)	47 (20.3)	
** Self-employed**		359 (53.4)	290 (65.9)	69 (29.7)	
** Student**		62 (9.2)	6 (1.4)	56 (24.1)	
** Unemployed**		31 (4.6)	23 (5.2)	8 (3.5)	
**Monthly Income (× 10**^**3**^ **₦), Median (Q1, Q3)**	671	30 (10, 50)	30 (10, 60)	20 (15, 46)	0.002^W^
**Marital Status, n (%)**	672				< 0.001^F^
** Married**		469 (69.8)	323 (73.4)	146 (62.9)	
** Widowed**		32 (4.8)	30 (6.8)	2 (0.86)	
** Divorced**		5 (0.74)	4 (0.91)	1 (0.43)	
** Separated**		47 (7.0)	36 (8.2)	11 (4.7)	
** Single/Never Married**		119 (17.7)	47 (10.7)	72 (31.0)	
**Smoking, n (%)**	672				0.249^F^
** Current**		16 (2.4)	10 (2.3)	6 (2.6)	
** Past**		26 (3.9)	21 (4.8)	5 (2.2)	
** Never**		630 (93.7)	409 (92.9)	221 (95.3)	
**Smoking (Past or Current), n (%)**		42 (6.3)	31 (7.1)	11 (4.7)	0.315^F^
**Alcohol Use, n (%)**	670				0.004^F^
** Current**		36 (5.4)	23 (5.2)	13 (5.6)	
** Past**		54 (8.1)	46 (10.5)	8 (3.5)	
** Never**		580 (86.6)	370 (84.3)	210 (90.9)	
**Alcohol Use (Past or Current), n (%)**	670	90 (13.4)	69 (15.7)	21 (9.1)	0.017^F^
**Physical Exercise, n (%)**	672	73 (10.9)	42 (9.6)	31 (13.4)	0.151^F^
**ART Duration (years), Median (Q1, Q3)**	439		12 (7, 15)		
**CD4 cell count/μL, Median (Q1, Q3)**	435		549 (403, 737)		
**WHO stage, n (%)**	439				
** I**			408 (92.9)		
** II**			28 (6.4)		
** III**			2 (0.46)		
** IV**			1 (0.23)		
**ART Regimen, n (%)**	439				
** TDF + 3TC (or FTC) + EFV**			10 (2.3)		
** TDF + 3TC (or FTC) + DTG**			344 (78.4)		
** AZT + 3TC + NVP (or EFV)**			1 (0.23)		
** ABC + 3TC + EFV**			5 (1.1)		
** AZT + 3TC + LPV/r**			15 (3.4)		
** AZT + 3TC + ATV/r**			11 (2.5)		
** TDF + 3TC + ATV/r**			29 (6.6)		
** TDF + 3TC + LPV/r**			24 (5.5)		
**Regimen Line, n (%)**	439				
** 1st Line**			360 (82.0)		
** 2nd Line**			79 (18.0)		
**Log10 Plasma HIV RNA copies/ml, Mean (SD)**	430		1.3 (1.3, 1.5)		
**Viral Load Category, n (%)**	430				
** < 20**			291 (67.7)		
** 20–49**			60 (13.9)		
** 50–99**			19 (4.4)		
** 100–199**			14 (3.3)		
** 200–399**			9 (2.1)		
** 400–999**			4 (0.9)		
** > = 1000**			33 (7.7)		

^w^Wilcoxon ^F^Fisher's

*Q1* 25th percentile, *Q3* 75th percentile *N* Number of participants, *SD* Standard deviation

*PLHIV* Persons living with HIV*, PWoH* Persons without HIV

About 49.7% of this cohort revealed adding salt on table to their food and this was similar among the PLHIV and PWoH (*P* = 0.289) (Appendix 1). The median fruit intake was 3 and 2 servings per week among the PLHIV and PWoH respectively (*P* = 0.016). Median vegetable intake was 4 servings per week, not significantly different by HIV status (*P* = 0.089) (Appendix 1).

The median hemoglobin level of participants was 13 g/dl, similar for both PLHIV and PWoH (*P* = 0.515). Using the Beck’s depression inventory, about 1.3% of the participants had scores indicative of depression, and this did not differ statistically by HIV status (*P* = 0.157) (Appendix 1).

The median duration on ART in this cohort was 12 years, with 82% on Nigeria’s first line (non-protease) regimen, and overwhelmingly transitioned to a DTG-based combination. About 92.8% were virally suppressed, a similar proportion were in WHO clinical stage I, and median CD4 count was 549 cells/µL (Table 1).

### Prevalence of metabolic syndrome and component disorders

**Fig. 1:**
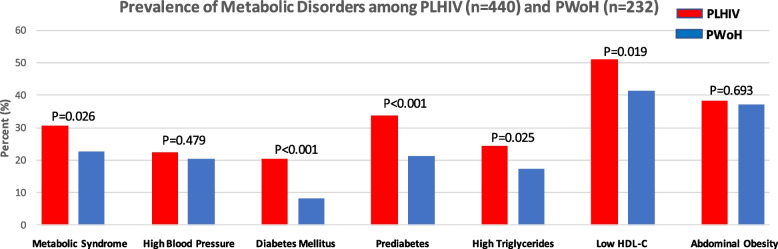
Prevalence of Metabolic Disorders among Persons Living with and without HIV Key: PLHIV: persons living with HIV; PWoH: persons without HIV; HDL-C: High density lipoprotein cholesterol; N: number of participants. Chi-square test was used to compare prevalence estimates

**Fig. 2:**
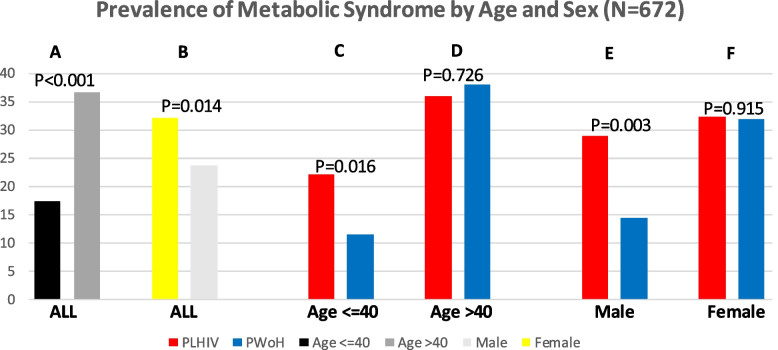
Prevalence of Metabolic Syndrome among Persons Living with and without HIV Stratified by Age and Sex **A** Prevalence of MetS among participants aged <  = 40 years and those > 40 years; **B** Prevalence of MetS in female and male participants; **C** Prevalence of MetS in PLHIV and PWoH among only participants aged <  = 40 years; **D** Prevalence of MetS in PLHIV and PWoH among only participants aged > 40 years; **E** Prevalence of MetS in PLHIV and PWoH among only male participants; **F** Prevalence of MetS in PLHIV and PWoH among only female participants Key: PLHIV: persons living with HIV; PWoH: persons without HIV; N: number of participants. Chi-square test was used to compare prevalence estimates

Overall, 30.7% (95% CI: 26.4%, 35.2%) of the PLHIV and 22.8% (95% CI: 17.6%, 28.8%) of the PWoH had MetS (*P* = 0.026) (Table 2). While about 10.3% of the participants declared they were previously diagnosed with hypertension (Appendix 1), up to 22.3% (95% CI: 18.5%, 26.5%) were found to have high blood pressure during assessment among the PLHIV, with no statistical differences by HIV status (Table 2). However, the PLHIV reported higher family history of hypertension (*P* = 0.004) (Appendix 1). Similarly, around 2.5% and 1.3% of the PLHIV and PWoH, respectively, were previously diagnosed with diabetes mellitus (*P* = 0.003) (Appendix 1). Following laboratory assessment, largely driven by HbA1c results, up to 20.5% (95% CI: 16.8%, 24.5%) of the PLHIV and 8.2% (95% CI: 5.0%, 12.5%) of the PWoH fulfilled the diagnostic criteria for diabetes mellitus (*P* < 0.001) (Table 2). Closely mirroring this pattern, 33.8% (95% CI: 29.5%, 38.5%) and 21.1% (95% CI: 16.1%, 26.9%) of the PLHIV and PWoH, respectively, had prediabetes (*P* < 0.001). An estimated 38.4% (95% CI: 33.8%, 43.1%) and 42.6% (95% CI: 38.0%, 47.5%) of the PLHIV had abdominal obesity, using waist circumference and waist-to-hip ratio measures respectively (Table 2). Utilizing BMI criteria, 52% of the participants were overweight or obese (Appendix 1). These anthropometric measures did not differ by HIV status. Around 24.5% (95% CI: 20.5%, 28.8%) of the PLHIV and 17.2% (95% CI: 12.6%, 22.7%) of the PWoH had high triglyceride levels (*P* = 0.025). Similarly, 51.1% (95% CI: 46.4%, 55.9%) and 41.4% (95% CI: 35.0%, 48.0%) of the PLHIV and PWoH, respectively, had low HDL-C (*P* = 0.019) (Fig. 1). The prevalence of MetS was higher among females as compared to males (32.2% vs 23.7%; *P* = 0.014) and among older as compared to younger participants (36.6% vs 17.3%; *P* < 0.001)) in pooled analysis of PLHIV and PWoH data (Fig. 2 charts A and B).

**Table 2:** Prevalence of Metabolic Syndrome and Component Disorders among Participants with and without HIV

Metabolic Condition	PLHIV				PWoH			
	**N**	**Estimate**	**95% CI**		**N**	**Estimate**	**95% CI**	
**Metabolic Syndrome**	440	30.7%	26.4%	35.2%	232	22.8%	17.6%	28.8%
**High Blood Pressure**	440	22.3%	18.5%	26.5%	232	20.3%	15.3%	26.0%
**Diabetes Mellitus**	440	20.5%	16.8%	24.5%	232	8.2%	5.0%	12.5%
**Prediabetes**	440	33.8%	29.5%	38.5%	232	21.1%	16.1%	26.9%
**Abdominal Obesity (WC)**	438	38.4%	33.8%	43.1%	232	37.1%	30.8%	43.6%
**Abdominal Obesity (WHR)**	438	42.6%	38.0%	47.5%	232	40.1%	33.7%	46.7%
**High Triglycerides**	437	24.5%	20.5%	28.8%	232	17.2%	12.6%	22.7%
**Low HDL-C**	437	51.1%	46.4%	55.9%	232	41.4%	35.0%	48.0%

*N *Number of participants, *PLHIV *Persons living with HIV, *PWoH *Persons without HIV, *CI *Confidence interval, *WC *Waist circumference, *WHR *Waist-to-hip ratio, *HDL-C *High density lipoprotein cholesterol

### Association of HIV with metabolic syndrome and component disorders

The odds of having the composite MetS were significantly higher among the PLHIV as compared to the PWoH in univariable analysis (OR: 1.53 [95% CI: 1.05, 2.2]; *P* = 0.026). This association failed to reach statistical significance following adjustment for age and sex (Table 3). The association between HIV and MetS appears to differ by strata of age and sex with significant interaction terms. Statistical significance was observed in univariable analyses among males (*P* = 0.003) but not females (*P* = 0.915) and among those aged 40 years or less (*P* = 0.016) but not among older participants (*P* = 0.726) (Fig. 2 charts C-F).

**Table 3:** Logistic regression for metabolic syndrome disorders comparing persons living with to those without HIV

Dependent Variable	N	Univariable				^a^Multivariable			
		**OR**	**95% CI**		***P*-value**	**OR**	**95% CI**		***P*-value**
**Metabolic Syndrome**	669	1.53	1.05	2.20	0.026	1.14	0.77	1.69	0.522
**High Blood Pressure**	671	1.15	0.78	1.71	0.479	0.76	0.49	1.16	0.199
**Diabetes Mellitus**	671	2.87	1.70	4.84	< 0.001	2.88	1.69	4.94	< 0.001
**Prediabetes**	671	1.95	1.34	2.84	< 0.001	1.70	1.16	2.50	0.007
**Abdominal Obesity (WC)**	669	1.07	0.77	1.49	0.693	0.80	0.55	1.16	0.239
**Abdominal Obesity (WHR)**	669	1.13	0.81	1.56	0.475	0.86	0.60	1.23	0.417
**High Triglycerides**	669	1.60	1.06	2.40	0.025	1.23	0.80	1.88	0.349
**Low HDL**	669	1.47	1.07	2.03	0.019	1.57	1.12	2.20	0.009

*N* Number of participants, *OR* Odds ratio, *CI* Confidence interval, *WC* Waist circumference, *WHR* Waist-to-hip ratio,

*HDL-C* High density lipoprotein cholesterol, *PLHIV* Persons living with HIV, *PWoH* Persons without HIV

^a^Adjusted for age and sex

In a multivariable logistic regression analysis adjusting for age and sex, the odds of elevated blood pressure were lower among the PLHIV (OR: 0.76 [95% CI: 0.49, 1.16]; *P* = 0.199) although not statistically significant. The odds of having diabetes mellitus and prediabetes were higher among the PLHIV as compared to the PWoH (OR: 2.88 [95% CI: 1.69, 4.94]; *P* < 0.001 and 1.7 [95% CI: 1.16, 2.5]; *P* = 0.007, respectively) (Table 3). Similarly, the odds of having low HDL-C were higher among the PLHIV (OR: 1.57 [95% CI: 1.12, 2.2]; *P* = 0.009). For hypertriglyceridemia, the odds were statistically significantly higher among the PLHIV only in univariable analysis (OR: 1.6 [95% CI: 1.06, 2.4]; *P* = 0.025). No statistically significant differences were observed for abdominal obesity between the PLHIV and PWoH in both univariable and multivariable analyses (Table 3).

### Characteristics associated with metabolic syndrome and component disorders

#### Metabolic syndrome

HIV, lower educational level, and ART duration were associated with metabolic syndrome only in univariable analysis. Independent associations were observed for older age (*P* < 0.001), female sex (*P* < 0.001), family history of hypertension (*P* = 0.013), family history of diabetes mellitus (*P* < 0.001), past alcohol use as compared to never use (*P* = 0.015), and higher frequency of fruit (*P* = 0.009) or vegetable (*P* = 0.008) consumption (Table 4).

**Table 4:** Characteristics associated with metabolic syndrome

Characteristic	N	Univariable				Multivariable			
		**OR**	**95% CI**		***P*-value**	**OR**	**95% CI**		***P*-value**
**PLHIV vs PWoH**	669	1.52	1.05	2.20	0.026	1.04	0.69	1.58	0.853
**Age**	669	1.06	1.04	1.07	< 0.001	1.07	1.05	1.09	< 0.001
**Education Primary vs Tertiary**	669	1.70	1.10	2.65	0.018	1.27	0.76	2.12	0.558
**Education Secondary vs Tertiary**	669	1.33	0.91	1.97	0.146	1.22	0.79	1.87	0.701
**Female vs Male**	669	1.55	1.10	2.18	0.012	2.73	1.78	4.21	< 0.001
**Family History of Hypertension**	669	2.14	1.50	3.07	< 0.001	1.69	1.12	2.55	0.013
**Family History of Diabetes**	669	2.98	2.01	4.44	< 0.001	2.69	1.70	4.26	< 0.001
**Vegetable Frequency**	669	1.13	1.04	1.23	0.004	1.14	1.03	1.25	0.008
**Fruit Frequency**	669	1.15	1.06	1.25	0.001	1.14	1.03	1.26	0.009
**Alcohol Use (Past or Current)**	669	1.79	1.13	2.85	0.013	1.97	1.14	3.39	0.015
**ART Duration**	439	1.06	1.01	1.10	0.015	1.02	0.97	1.07	0.462

*N* Number of participants, *OR* Odds ratio, *CI* Confidence interval, *ART* Antiretroviral treatment, *PLHIV* Persons living with HIV, *PWoH* Persons without HIV

### High blood pressure

In univariable analyses, older age, lower level of education, diabetes mellitus, abdominal obesity, high triglycerides, smoking, history of alcohol use, family history of hypertension, and physical exercise were associated with high blood pressure. Among the PLHIV, longer duration on ART was also associated with high blood pressure. Of these, increasing age (*P* < 0.001), lower level of education (*P* = 0.035), diabetes mellitus (*P* = 0.020), abdominal obesity (*P* < 0.001), high triglycerides (*P* = 0.039), family history of hypertension (*P* = 0.004) and physical exercise (*P* = 0.006) showed independent associations in adjusted analysis (Appendix 2).

### Diabetes mellitus

In both univariable and multivariable analyses, HIV (*P* < 0.001), high blood pressure (*P* = 0.049), lower hemoglobin levels (*P* < 0.001), and family history of diabetes (*P* = 0.002) were significantly associated with diabetes mellitus. Depression tended to be associated with a higher likelihood of having diabetes mellitus though not reaching statistical significance (*P* = 0.074) (Appendix 3).

### Prediabetes

HIV, older age, female sex, high triglycerides, low HDL-C, family history of hypertension, family history of diabetes, and higher vegetable use frequency were associated with prediabetes in univariable analyses. Following adjustment, HIV (*P* = 0.016), older age (*P* = 0.008), female sex (*P* = 0.005) and family history of diabetes (*P* = 0.033) remained independently associated with prediabetes (Appendix 4).

### Hypertriglyceridemia

For both univariable and multivariable analyses, older age (*P* = 0.002), low HDL-C (*P* < 0.001), abdominal obesity (*P* = 0.016), and high blood pressure (*P* = 0.029), were associated with hypertriglyceridemia. HIV, male sex, family history of diabetes, higher hemoglobin, ever smoked or used alcohol were only associated in univariable analysis. Among the PLHIV, higher HIV RNA viral load (*P* = 0.024) and use of protease regimen (*P* = 0.019) were independently associated with hypertriglyceridemia. Duration on ART tended towards showing higher association with high triglycerides in univariable analysis but did not reach statistical significance (*P* = 0.066 (Appendix 5).

### Low HDL-C

HIV (*P* = 0.046), female sex (*P* < 0.001), high triglycerides (*P* < 0.001), and absence of abdominal obesity (*P* < 0.001), were independently associated with low HDL-C levels. For PLHIV, those in WHO clinical stage 2 or higher as compared to stage 1 showed a trend towards significance for independent association with low HDL-C (*P* = 0.072) (Appendix 6).

### Abdominal obesity

Independent associations with abdominal obesity were observed for older age (*P* < 0.001), female sex (*P* < 0.001), high blood pressure (*P* < 0.001), low HDL-C (P < 0.001), high triglycerides (*P* = 0.013), family history of hypertension (*P* < 0.011) or diabetes (*P* = 0.036), higher income (*P* = 0.029), alcohol use (*P* = 0.002), and higher fruit use frequency (*P* = 0.026). Lower education was associated only in unadjusted analysis. Among the PLHIV, CD4 count below 350 cells/µL as compared to higher levels, and higher viral load by a trend, were associated with lower likelihood of having abdominal obesity in univariable analyses. In contrast, ART duration was associated with greater likelihood, also in unadjusted analysis (Appendix 7).

## Discussion

In this cohort, the prevalence of MetS was 30.7% and 22.8% among PLHIV and PWoH respectively. We found significantly higher prevalence of dysglycemia (prediabetes and diabetes mellitus) and dyslipidemia (low HDL-C and high triglycerides) among PLHIV as compared to PWoH.

The prevalence of MetS obtained for the PLHIV is towards the higher range of estimates reported by other studies from Nigeria [21–24], other African countries [10, 25], and the pooled global estimates [11]. This is consistent with expectations, as our cohort is composed exclusively of treatment-experienced individuals. Previous studies that compared treatment-naïve to treatment-experienced PLHIV found significantly higher prevalence among the latter [24]. Therefore, such relatively high prevalence may be a further indication of the link between antiretroviral medications and metabolic disorders, which may be mediated through direct effects of ART or via restoration of health among persons otherwise genetically predisposed [7].

Similar to other reports for both PLHIV and the general population, we found strong associations of MetS with older age and female sex [11] as well as alcohol use [26]. Interestingly, our analyses also found direct correlations between MetS and higher frequency of fruit and vegetable intake. While there are few studies reporting a similar finding [27, 28], and many others showing no association [29], the predominant evidence indicates an inverse relationship, with higher vegetable and fruit intake being associated with lower risk of developing MetS. Therefore, our finding may be a manifestation of the phenomenon of prevalence-incidence bias, known to occur in cross-sectional studies, due to differential survival between comparison groups in the source population. Alternatively, it may also be due to reverse causality, implying that individuals might be adopting a healthier lifestyle, in this case higher fruit and vegetable intake, following diagnoses of metabolic disorders.

Among the PLHIV, ART duration showed significant association in univariable analysis, though the pattern remained similar following adjustment. Other studies also reported correlations with duration on ART or time since HIV diagnosis, in addition to lower CD4 count, higher viral load, and protease inhibitor regimen use [11]. Our estimates of association for the latter three were in a similar direction though not statistically significant. Overall, the patterns of these associations indicate that poorly controlled HIV disease likely contributes to the development of the metabolic syndrome. Thus, while the predominate pathway to metabolic syndrome might be through restoration of health following ART, persisting viremia and its associated chronic inflammation as well as the toxic effects of some antiretroviral medications may be additional factors in this rather complex causal relationship.

The prevalence of 22.3% for high blood pressure among PLHIV in our cohort is similar to some reports from Nigeria [24, 30, 31] as well as global pooled estimates [32, 33]. We did not find a statistically significant difference in prevalence between the PLHIV and PWoH in our cohort. Other studies reported variable findings regarding the association between HIV and high blood pressure [34]. Such differences might be due to differences in the characteristics of participants included, their HIV disease status, and possible methodological limitations. Among the HIV-related factors we explored, only duration on ART correlated with high blood pressure, and only in unadjusted analysis. Other studies found associations with ART duration too, but also lower CD4 nadir, unsuppressed viremia, and protease inhibitor regimen [35–37]. Our inability to observe similar associations might be due to our cohort being much healthier with respect to HIV disease control. Like other reports linking high blood pressure with tobacco smoking and alcohol use[38, 39], our analyses found similar associations, though only in univariable models. Expectedly, we did find strong correlations with older age and family history which are non-modifiable risk factors for hypertension as widely reported [32, 40]. Consistent with many reports for PLHIV and the general population, we found significant associations with abdominal obesity, diabetes mellitus and high triglycerides [41–44]. The observed correlations between these disorders and high blood pressure is further indication of their significant level of co-occurrence and likely interconnected causal relationship [45]. Establishing precise mechanistic pathways involved in this will be invaluable towards identifying holistic preventative and therapeutic interventions.

Our estimated prevalence of 20.5% for diabetes mellitus among the PLHIV is also towards the high range of estimates reported by other studies [46–51]. Similar to many reports, we found significantly higher prevalence of diabetes among the PLHIV [49, 50, 52–54]. However, a few studies [55] did report lower prevalence estimates while others failed to find differences by HIV status [56–58]. Of the traditional risk factors reported by other studies like age, sex, and other co-morbid conditions [46, 50, 52, 59], we only found significant associations with high blood pressure and family history. Other studies found associations with HIV-related factors like lower CD4 count, higher viral load, and protease inhibitor regimen [53, 60, 61].

The prevalence of 33.8% for prediabetes among the PLHIV was also on the high side of the range reported from other African countries (19–47%) [46, 50]. Like other reports, our analyses found significant associations with HIV, older age, and family history of diabetes mellitus [49, 50, 62].

As previously reported [50, 63, 64], and similar to our observation for high blood pressure, the majority of participants determined to have diabetes mellitus in this cohort were unaware of their diagnosis. This further underscores the need to intensify efforts towards regular and frequent screening of diabetes mellitus and hypertension especially among PLHIV, but also in the general population, as a cornerstone of comprehensive primary prevention strategies for these disorders.

The prevalence of 24.5% and 51.1% that we obtained for high triglycerides and low HDL-C, respectively, are comparable to other reports from Nigeria [65, 66], other African countries [67, 68], and elsewhere [69, 70]. However, some studies did report lower [71] and higher [72, 73] prevalence estimates as well. Like many earlier studies, we found significant associations between HIV and low HDL-C or high triglycerides, though only in univariable analysis for the latter [69, 74–76]. Furthermore, our analyses found independent associations of high triglycerides with older age, abdominal obesity, high blood pressure, low HDL-C, and tobacco smoking as reported earlier [77–79]. Though only in unadjusted analyses, we did observe correlations of high triglycerides with higher income, higher hemoglobin, and higher fruit use frequency, which are suggestive of a likely association with higher socioeconomic status as previously reported [80].

Similar to other reports for HIV-related factors [76, 79], we found strong independent association of high triglycerides with higher viral load and use of protease inhibitor regimen, but also with duration on ART in univariable analyses. For low HDL-C, female sex, older age, and abdominal obesity showed independent associations as in earlier reports [77, 79, 81]. We also found an association with higher WHO clinical stage among the PLHIV in unadjusted analysis, in addition to a trend towards significance after adjustment, which likely reflects reported associations with higher viral load and lower CD4 count in other studies [74, 79]. Overall, these findings seem to suggest that poorly controlled HIV disease may have a significant causal relationship with dyslipidemia.

The prevalence estimates we obtained for abdominal obesity using waist circumference (WC) and waist-to-hip ratio (WHR) are comparable (38.4% and 42.6%). This provides some justification for using the European rather than the American cut-off points for WC in this study as recommended for African populations [11, 82]. Nevertheless, studies are needed to determine appropriate cut-offs for Nigeria and other African populations. As expected, the prevalence estimates for overweight/obesity and obesity using the BMI criteria (52.6% and 17.6%) differ significantly. The higher concordance among the proxy measures of central adiposity (WC and WHR) and their superiority in predicting cardiometabolic outcomes when compared to the BMI have been previously documented [83, 84]. Compared to other reports, our prevalence estimates for these adiposity measures are higher than many [85–89], similar to others [24, 90–95] and lower than some [96–99]. Unlike some earlier studies, we did not find significant differences by HIV status [99–101]. However, consistent with other reports, we found independent associations with traditional factors like older age, female sex, high blood pressure, dyslipidemia, alcohol use and higher income [93, 95, 102–104], in addition to higher CD4 count and ART duration in unadjusted analysis among the PLHIV [102]. These essentially reflect and strengthen our knowledge on the role of some demographic characteristics, metabolic comorbidity, antiretroviral agents as well as markers of restored health and economic wellbeing [105] in the pathogenesis of obesity, and may represent focus areas for targeted inventions.

The high prevalence estimates for the different adiposity measures in this study reflect the rising trend of overweight and obesity among PLHIV which generally mimics what is observed in the general population [102]. Interestingly, our estimates closely mirror the pooled estimates for the general population in Nigeria and other African countries [106]. Such rising trends are quite alarming because of the implications for multimorbidity and downstream adverse cardiovascular outcomes [107].

The significance of the metabolic syndrome cluster is generally attributed to its strong link with a heightened risk for diabetes mellitus, atherosclerotic and non-atherosclerotic cardiovascular disease, and all-cause mortality [9]. Although the risk for adverse outcomes was shown to correlate with the number of disorders, it remains unclear whether the effects of the combination are additive or synergistic [108]. It is also unclear as to whether the component disorders represent distinct pathological processes, or they are manifestations of a common pathogenic mechanism. Nonetheless, major pathways considered pivotal in the initiation and progression of this multiple risk factor cluster include visceral adiposity, insulin resistance, neurohumoral dysregulation and chronic inflammation [109]. Lifestyle and environmental factors are believed to also play important roles or even serve as primary triggers for this complex clinicopathological phenomenon.

Whereas HIV-related factors have been shown to be associated with cardiometabolic disorders, their precise role in pathogenesis remains unknown. Some of the mechanistic pathways thought to underlie the accelerated occurrence of such disorders in HIV include persistent viremia, microbial translocation, gut microbiome dysbiosis, antiretroviral drug toxicity, chronic inflammation as well as dysregulated adaptive and innate immune system [110]. Further research is needed to characterize the differential contributions of these factors in pathogenesis and the interventions needed to address them.

Antiretroviral drug toxicity is a key modifiable risk factor for the emergence and progression of MetS disorders. However, its contribution has declined following the introduction of newer agents with better safety profile [111, 112]. During the early era of ART, first generation protease inhibitors (e.g. indinavir, nelfinavir and saquinavir) and thymidine analogues (e.g. stavudine, didanosine, and zidovudine) have been notoriously linked to the development of lipodystrophy and the metabolic syndrome phenotype [113]. The mechanistic processes involved are not fully known but believed to be principally mediated through mitochondrial dysfunction, lipodystrophy, induction of a pro-inflammatory milieu, and insulin resistance [7]. Central in the pathogenesis is an altered expression of pro-inflammatory cytokines (increased tumor necrosis factor alpha [TNF-α], interleukin-6 [IL-6], and interleukin-1 [IL-1], as well as decreased adipokines). These lead to changes in key metabolic pathways, including inhibition of lipoprotein lipase, peroxisome proliferator-activated receptor-gamma (PPARꝩ) and glucose transporter (GLUT4), resulting in dyslipidemia, weight changes and dysglycemia [113, 114].

Despite improvement in the overall safety of newer generation antiretroviral drugs, metabolic adverse effects persist, albeit in relatively less pronounced forms. The recent transition from NNRTI-based to INSTI-based regimen has been shown to be associated with significant weight gain and other adverse metabolic changes [115, 116]. Emerging studies suggest that INSTIs, especially DTG, may increase the expression of pro-inflammatory cytokines like IL-6 while repressing adiponectin and leptin expression, with the potential combined effect of weight gain and metabolic dysregulation [117]. Overall, antiretroviral metabolic toxicity tends to be a class effect, though some agents may be associated with worse manifestations than others. Furthermore, the severity of this adverse effect was reported to strongly correlate with demographic and clinical characteristics such as age, sex, duration on treatment, body mass index, and immune status [113]. Therefore, continued clinical vigilance is required in antiretroviral therapy, especially during regimen selection at treatment initiation, but also for switch and substitution decisions. Clinicians must consider the overall risk profile of patients, through comprehensive clinical evaluation, in addition to instituting appropriate management modalities to prevent and control metabolic disorders [118].

This study has some limitations. First, as a cross-sectional analysis, we were unable to assess temporal sequence and the impact of longitudinal changes in risk factors on our outcomes. Second, our sex-balanced recruitment may have lowered the actual prevalence estimates for the MetS because of the preponderance of women among PLHIV in our study setting and the known higher burden among women. However, we found miniscule difference (less than 0.1%) with sex-weighted prevalence obtained using projected sex proportions for the study setting. Third, the frequency matching for age implemented among the controls fell short of achieving balance for age in our comparison groups. Therefore, to address potential residual confounding, we adjusted for age and sex in our analysis models. Fourth, due to lack of fasting blood glucose (FBS) data, which is recommended by the NCEP/ATPIII criteria for the definition of dysglycemia, we used HbA1c and random blood glucose instead. Thus, our assessment of diabetes and prediabetes were largely driven by results of HbA1c test which is known to underestimate these conditions among PLHIV. However, we expect this to be minimal because most of our participants had normal hemoglobin levels and only a small proportion were on zidovudine-based regimen. Moreover, some studies did report high concordance between criteria using FBS and those using HbA1c for the determination of MetS [119, 120]. Therefore, while HbA1c may have low sensitivity for detecting dysglycemia as a solitary condition [121], this may not be the case for the MetS cluster, suggesting likely buffering effects within this multimorbidity complex, and further demonstrating the clinical and pathological relevance of the syndrome.

Notable strengths of this study include a relatively large sample size for adequate exploration of associations and determination of robust estimates with high precision. Unlike many other studies, we recruited PWoH matched by age and sex for effective comparison and estimation of HIV-related measures of association. Furthermore, our PLHIV had a broad range for duration on ART. This enabled meaningful assessment of its association with the metabolic conditions explored, in addition to de-confounding other associations, in lieu of ART-naïve individuals in this study.

## Conclusion

In this study of ART-experienced individuals, we found prevalence estimates for metabolic syndrome and its components towards the high range of estimates reported in earlier studies. This may be partly due to our participants’ lengthy exposure to antiretroviral drugs, with the vast majority already transitioned to the INSTI-based regimen known to be associated with adverse metabolic outcomes. We found significantly higher prevalence of dysglycemia and dyslipidemia among the PLHIV, in addition to observing independent associations with traditional risk factors. Overall, our results, like those of previous reports, clearly underscore the excess metabolic risks experienced by PLHIV. This might portend downstream adverse cardiovascular outcomes and other poor health sequelae in an already overburdened and disadvantaged segment of the population. While further research is needed to understand the biological and behavioral bases of these risks and the interventions required to address them, efforts need to be intensified towards implementing known preventive and therapeutic strategies. Such would include comprehensive screening for these disorders, appropriate linkage to care and treatment, as well as deliberate measures towards achieving and sustaining satisfactory therapeutic control. In this regard, there is a glaring need for more implementation research to identify optimal strategies for integrating prevention and management modalities of MetS disorders in HIV treatment settings.

## Supplementary Information

**Additional file 1: Appendix 1.** Demographic and clinical characteristics. **Appendix 2.** Characteristics associated with high blood pressure. **Appendix 3.** Characteristics associated with diabetes mellitus. **Appendix 4.** Characteristics associated with prediabetes. **Appendix 5.** Characteristics associated with high triglycerides. **Appendix 6.** Characteristics associated with low HDL-C. **Appendix 6.** Characteristics associated with low HDL-C. **Appendix 7.** Characteristics associated with abdominal obesity.

## Data Availability

The dataset used in this study is available upon reasonable request to the corresponding author.

## Abbreviations

ART: Antiretroviral therapy/treatment
BMI: Body mass index
DTG: Dolutegravir
HDL-C: High density lipoprotein cholesterol
INSTI: Integrase strand transfer inhibitors
MetS: Metabolic syndrome
NCD: Non-communicable disease
NCEP ATP III: National Cholesterol Education Program Adult Treatment Panel (3^rd^ report)
NNRTI: Non-nucleoside reverse transcriptase inhibitors
PEPFAR: United States President’s Emergency Plan for AIDS Relief
PLHIV: Persons living with HIV
PWoH: Persons without HIV
STEPS: Stepwise approach to non-communicable disease surveillance
WHO: World Health Organization
