# Supplementary material for: Prevalence and characteristics of metabolic syndrome and its components among adults living with and without HIV in Nigeria: a single-center study
Source: BMC Endocr Disord. 2023 Jul 28;23:160. doi: 10.1186/s12902-023-01419-x (PMC10375691; doi:10.1186/s12902-023-01419-x)
Supplement: Supplementary file 1 — Additional file 1: Appendix 1. Demographic and clinical characteristics. Appendix 2. Characteristics associated with high blood pressure. Appendix 3. Characteristics associated with diabetes mellitus. Appendix 4. Characteristics associated with prediabetes. Appendix 5. Characteristics associated with high triglycerides. Appendix 6. Characteristics associated with low HDL-C. Appendix 6. Characteristics associated with low HDL-C. Appendix 7. Characteristics associated with abdominal obesity. [file 12902_2023_1419_MOESM1_ESM.docx]

| **Appendix 1: Demographic and clinical characteristics** | | | | | | | | | | | |  | |  |
| --- | --- | --- | --- | --- | --- | --- | --- | --- | --- | --- | --- | --- | --- | --- |
|  |  |  |  |  |  |  |  |  |  |  |  |  | |  |
| **Characteristic** | | | **N** | **All**  **672** | | | **PLHIV**  **440** | | **PWoH**  **232** | | | **P-value** | |  |
| **Age (years), Median (Q1, Q3)** | | | 672 | 42 (34, 50) | | | 45 (36, 52) | | 40 (26, 46.5) | | | < 0.001^W^ | |  |
| **Gender, Female n (%)** | | | 672 | 335 (49.9) | | | 222 (50.5) | | 119 (51.3) | | | 0.686^F^ | |  |
| **Education, n (%)** | | | 672 |  | | |  | |  | | | < 0.001^F^ | |  |
| **None/Primary** | | |  | 139 (20.7) | | | 105 (23.9) | | 34 (14.6) | | |  | |  |
| **Junior/Senior Secondary** | | |  | 236 (35.1) | | | 163 (37.0) | | 73 (31.5) | | |  | |  |
| **Tertiary** | | |  | 297 (44.2) | | | 172 (39.1) | | 125 (53.9) | | |  | |  |
| **Occupation, n (%)** | | | 672 |  | | |  | |  | | | < 0.001^F^ | |  |
| **Civil Service/Governmental** | | |  | 152 (22.6) | | | 100 (22.7) | | 52 (22.4) | | |  | |  |
| **Non-governmental** | | |  | 68 (10.1) | | | 21 (4.8) | | 47 (20.3) | | |  | |  |
| **Self-employed** | | |  | 359 (53.4) | | | 290 (65.9) | | 69 (29.7) | | |  | |  |
| **Student** | | |  | 62 (9.2) | | | 6 (1.4) | | 56 (24.1) | | |  | |  |
| **Unemployed** | | |  | 31 (4.6) | | | 23 (5.2) | | 8 (3.5) | | |  | |  |
| **Monthly Income (x10^3^ ₦), Median (Q1, Q3)** | | | 671 | 30 (10, 50) | | | 30 (10, 60) | | 20 (15, 46) | | | 0.002^W^ | |  |
| **Marital Status, n (%)** | | | 672 |  | | |  | |  | | | < 0.001^F^ | |  |
| **Married** | | |  | 469 (69.8) | | | 323 (73.4) | | 146 (62.9) | | |  | |  |
| **Widowed** | | |  | 32 (4.8) | | | 30 (6.8) | | 2 (0.86) | | |  | |  |
| **Divorced** | | |  | 5 (0.74) | | | 4 (0.91) | | 1 (0.43) | | |  | |  |
| **Separated** | | |  | 47 (7.0) | | | 36 (8.2) | | 11 (4.7) | | |  | |  |
| **Single/Never Married** | | |  | 119 (17.7) | | | 47 (10.7) | | 72 (31.0) | | |  | |  |
| **Smoking, n (%)** | | | 672 |  | | |  | |  | | | 0.249^F^ | |  |
| **Current** | | |  | 16 (2.4) | | | 10 (2.3) | | 6 (2.6) | | |  | |  |
| **Past** | | |  | 26 (3.9) | | | 21 (4.8) | | 5 (2.2) | | |  | |  |
| **Never** | | |  | 630 (93.7) | | | 409 (92.9) | | 221 (95.3) | | |  | |  |
| **Alcohol Use, n (%)** | | | 670 |  | | |  | |  | | | 0.004^F^ | |  |
| **Current** | | |  | 36 (5.4) | | | 23 (5.2) | | 13 (5.6) | | |  | |  |
| **Past** | | |  | 54 (8.1) | | | 46 (10.5) | | 8 (3.5) | | |  | |  |
| **Never** | | |  | 580 (86.6) | | | 370 (84.3) | | 210 (90.9) | | |  | |  |
| **Physical Exercise, n (%)** | | | 672 | 73 (10.9) | | | 42 (9.6) | | 31 (13.4) | | | 0.151^F^ | |  |
| **Salt added on table, n (%)** | | | 672 |  | | |  | |  | | | 0.289^F^ | |  |
| **Never** | | |  | 244 (36.3) | | | 153 (34.8) | | 91 (39.2) | | |  | |  |
| **Rarely** | | |  | 14 (2.1) | | | 12 (2.7) | | 2 (0.86) | | |  | |  |
| **Sometimes** | | |  | 56 (8.3) | | | 34 (7.7) | | 22 (9.5) | | |  | |  |
| **Often** | | |  | 24 (3.6) | | | 18 (4.1) | | 6 (2.6) | | |  | |  |
| **Always** | | |  | 334 (49.7) | | | 223 (50.7) | | 111 (47.8) | | |  | |  |
| **Fruit Frequency per week, Median (Q1, Q3)** | | | 672 | 2 (2, 4) | | | 3 (2, 4) | | 2 (2, 4) | | | 0.016^W^ | |  |
| **Vegetable Frequency per week, Median (Q1, Q3)** | | | 672 | 4 (2, 5) | | | 4 (2, 5) | | 3 (2, 4) | | | 0.089^W^ | |  |
| **Self-reported conditions** | | |  |  | | |  | |  | | |  | |  |
| **Hypertension, n (%)** | | | 671 | 69 (10.3) | | | 49 (11.1) | | 20 (8.7) | | | 0.587^F^ | |  |
| **Prediabetes, n (%)** | | | 671 | 7 (1.0) | | | 4 (0.91) | | 1 (1.3) | | | 0.713^F^ | |  |
| **Diabetes Mellitus, n (%)** | | | 671 | 14 (2.1) | | | 11 (2.5) | | 3 (1.3) | | | 0.003^F^ | |  |
| **Family History of Hypertension, n (%)** | | | 672 | 194 (28.9) | | | 143 (32.5) | | 51 (22.0) | | | 0.004^F^ | |  |
| **Family History of Diabetes, n (%)** | | | 672 | 132 (19.6) | | | 90 (20.5) | | 42 (18.1) | | | 0.540^F^ | |  |
| **BMI Categories, n (%)** | | | 669 |  | | |  | |  | | | 0.863^F^ | |  |
| **Normal** | | |  | 294 (43.9) | | | 191 (43.7) | | 103 (44.4) | | |  | |  |
| **Underweight** | | |  | 27 (4.0) | | | 16 (3.7) | | 11 (4.7) | | |  | |  |
| **Overweight** | | |  | 229 (34.2) | | | 153 (35.0) | | 76 (32.8) | | |  | |  |
| **Obese** | | |  | 119 (17.8) | | | 77 (17.6) | | 42 (18.1) | | |  | |  |
| **Hemoglobin, Median (Q1, Q3)** | | | 670 | 13 (12, 14.2) | | | 13 (11.9, 14.2) | | 13 (12.1, 14.2) | | | 0.515^W^ | |  |
| **Beck's Depression Score, Median (Q1, Q3)** | | | 671 | 1 (1, 3) | | | 1 (1, 3) | | 1 (1, 2) | | | < 0.001^W^ | |  |
| **Depression (BDI >=14), n (%)** | | | 671 | 9 (1.3) | | | 8 (1.8) | | 1 (0.43) | | | 0.175^F^ | |  |
| **ART Duration (years), Median (Q1, Q3)** | | | 439 |  | | | 12 (7, 15) | |  | | |  | |  |
| **CD4 cell count/μl, Median (Q1, Q3)** | | | 435 |  | | | 549 (403, 737) | |  | | |  | |  |
| **WHO stage, n (%)** | | | 439 |  | | |  | |  | | |  | |  |
| **I** | | |  |  | | | 408 (92.9) | |  | | |  | |  |
| **II** | | |  |  | | | 28 (6.4) | |  | | |  | |  |
| **III** | | |  |  | | | 2 (0.46) | |  | | |  | |  |
| **IV** | | |  |  | | | 1 (0.23) | |  | | |  | |  |
| **ART Regimen, n (%)** | | | 439 |  | | |  | |  | | |  | |  |
| **TDF+3TC (or FTC) +EFV** | | |  |  | | | 10 (2.3) | |  | | |  | |  |
| **TDF+3TC (or FTC) +DTG** | | |  |  | | | 344 (78.4) | |  | | |  | |  |
| **AZT+3TC +NVP (or EFV)** | | |  |  | | | 1 (0.23) | |  | | |  | |  |
| **ABC +3TC+EFV** | | |  |  | | | 5 (1.1) | |  | | |  | |  |
| **AZT + 3TC + LPV/r** | | |  |  | | | 15 (3.4) | |  | | |  | |  |
| **AZT+ 3TC + ATV/r** | | |  |  | | | 11 (2.5) | |  | | |  | |  |
| **TDF +3TC + ATV/r** | | |  |  | | | 29 (6.6) | |  | | |  | |  |
| **TDF +3TC + LPV/r** | | |  |  | | | 24 (5.5) | |  | | |  | |  |
| **Regimen Line, n (%)** | | | 439 |  | | |  | |  | | |  | |  |
| **1st Line** | | |  |  | | | 360 (82.0) | |  | | |  | |  |
| **2nd Line** | | |  |  | | | 79 (18.0) | |  | | |  | |  |
| **Log10 Plasma HIV RNA copies/ml, Mean (SD)** | | | 430 |  | | | 1.3 (1.3, 1.5) | |  | | |  | |  |
| **Viral Load Category, n (%)** | | | 430 |  | | |  | |  | | |  | |  |
| **<20** | | |  |  | | | 291 (67.7) | |  | | |  | |  |
| **20-49** | | |  |  | | | 60 (13.9) | |  | | |  | |  |
| **50-99** | | |  |  | | | 19 (4.4) | |  | | |  | |  |
| **100-199** | | |  |  | | | 14 (3.3) | |  | | |  | |  |
| **200-399** | | |  |  | | | 9 (2.1) | |  | | |  | |  |
| **400-999** | | |  |  | | | 4 (0.9) | |  | | |  | |  |
| **>=1000** | | |  |  | | | 33 (7.7) | |  | | |  | |  |
| ^w^Wilcoxon ^F^Fisher's | | |  |  | | |  | |  | | |  | |  |
| Q1: 25th percentile; Q3: 75th percentile N: number of participants;SD: standard deviation; | | | | | | | | |  | | |  | |  |
| PLHIV: persons living with HIV; PWoH: persons without HIV | | | | | | | | |  | | |  | |  |
| **Appendix 2: Characteristics associated with high blood pressure** | | | | | | | | | | | | | | |
| **Characteristic** | **N** | **Univariable** | | | | | | **Multivariable** | | | | | | |
|  |  | **OR** | **95% CI** | | | **P-value** | | **OR** | | **95% CI** | | | **P-value** | |
| **PLHIV vs PWoH** | 671 | 1.15 | 0.78 | | 1.71 | 0.479 | | 0.68 | | 0.43 | 1.08 | | 0.104 | |
| **Age** | 671 | 1.07 | 1.05 | | 1.09 | < 0.001 | | 1.07 | | 1.04 | 1.09 | | < 0.001 | |
| **Education Primary vs Tertiary** | 671 | 2.64 | 1.63 | | 4.27 | < 0.001 | | 2.39 | | 1.37 | 4.16 | | 0.035 | |
| **Education Secondary vs Tertiary** | 671 | 1.92 | 1.24 | | 2.97 | 0.004 | | 2.01 | | 1.23 | 3.28 | | 0.235 | |
| **Diabetes Mellitus** | 671 | 1.76 | 1.12 | | 2.79 | 0.015 | | 1.87 | | 1.10 | 3.16 | | 0.020 | |
| **Abdominal Obesity** | 669 | 3.43 | 2.34 | | 5.03 | < 0.001 | | 2.54 | | 1.66 | 3.89 | | < 0.001 | |
| **High Triglycerides** | 669 | 2.37 | 1.58 | | 3.57 | < 0.001 | | 1.63 | | 1.03 | 2.59 | | 0.039 | |
| **Family History of Hypertension** | 671 | 2.29 | 1.56 | | 3.36 | < 0.001 | | 1.89 | | 1.22 | 2.93 | | 0.004 | |
| **Physical Exercise** | 671 | 1.81 | 1.07 | | 3.09 | 0.029 | | 2.47 | | 1.30 | 4.68 | | 0.006 | |
| **Smoking (Past or Current)** | 671 | 2.41 | 1.26 | | 4.63 | 0.008 | | 1.40 | | 0.63 | 3.14 | | 0.413 | |
| **Alcohol Use (Past or Present)** | 670 | 2.04 | 1.26 | | 3.32 | 0.004 | | 1.11 | | 0.61 | 2.01 | | 0.738 | |
|  |  |  |  | |  |  | |  | |  |  | |  | |
| **ART Duration** | 439 | 1.08 | 1.03 | | 1.14 | 0.002 | | 1.03 | | 0.97 | 1.09 | | 0.299 | |
|  |  |  |  | |  |  | |  | |  |  | |  | |
| N: number of participants; OR: odds ratio; CI: confidence interval; ART: antiretroviral treatment | | | | | | | | | |  |  | |  | |
| PLHIV: persons living with HIV; PWoH: persons without HIV; | | | | |  |  | |  | |  |  | |  | |

| **Appendix 3: Characteristics associated with diabetes mellitus** | | | | | | | | | |
| --- | --- | --- | --- | --- | --- | --- | --- | --- | --- |
| **Characteristic** | **N** | **Univariable** | | | | **Multivariable** | | | |
|  |  | **OR** | **95% CI** | | **P-value** | **OR** | **95% CI** | | **P-value** |
| **PLHIV vs PWoH** | 671 | 2.87 | 1.70 | 4.84 | < 0.001 | 2.75 | 1.61 | 4.69 | < 0.001 |
| **High Blood Pressure** | 671 | 1.76 | 1.12 | 2.79 | 0.015 | 1.63 | 1.00 | 2.64 | 0.049 |
| **Family History of Diabetes** | 671 | 2.17 | 1.37 | 3.44 | 0.001 | 2.19 | 1.35 | 3.56 | 0.002 |
| **Depression** | 671 | 4.24 | 1.12 | 16.04 | 0.034 | 3.42 | 0.89 | 13.19 | 0.074 |
| **Hemoglobin level** | 670 | 0.77 | 0.68 | 0.87 | < 0.001 | 0.78 | 0.69 | 0.89 | < 0.001 |
|  |  |  |  |  |  |  |  |  |  |
| N: number of participants; OR: odds ratio; CI: confidence interval; ART: antiretroviral treatment | | | | | | | |  |  |
| PLHIV: persons living with HIV; PWoH: persons without HIV; | | | |  |  |  |  |  |  |

| **Appendix 4: Characteristics associated with prediabetes** | | | | | | | | | |
| --- | --- | --- | --- | --- | --- | --- | --- | --- | --- |
| **Characteristic** | **N** | **Univariable** | | | | **Multivariable** | | | |
|  |  | **OR** | **95% CI** | | **P-value** | **OR** | **95% CI** | | **P-value** |
| **PLHIV vs PWoH** | 671 | 1.95 | 1.34 | 2.84 | < 0.001 | 1.62 | 1.10 | 2.41 | 0.016 |
| **Age** | 671 | 1.03 | 1.01 | 1.04 | < 0.001 | 1.02 | 1.01 | 1.04 | 0.008 |
| **Female vs Male** | 671 | 1.48 | 1.06 | 2.07 | 0.021 | 1.69 | 1.18 | 2.44 | 0.005 |
| **High Triglycerides** | 669 | 1.68 | 1.14 | 2.47 | 0.008 | 1.36 | 0.90 | 2.05 | 0.144 |
| **Low HDL-C** | 669 | 1.44 | 1.04 | 2.02 | 0.031 | 1.32 | 0.93 | 1.88 | 0.126 |
| **Family History of Hypertension** | 671 | 1.42 | 1.00 | 2.04 | 0.053 | 1.15 | 0.78 | 1.70 | 0.482 |
| **Family History of Diabetes** | 671 | 1.79 | 1.20 | 2.67 | 0.004 | 1.61 | 1.04 | 2.50 | 0.033 |
| **Vegetable Freq** | 671 | 1.08 | 1.00 | 1.18 | 0.054 | 1.09 | 1.00 | 1.18 | 0.054 |
|  |  |  |  |  |  |  |  |  |  |
| N: number of participants; OR: odds ratio; CI: confidence interval; ART: antiretroviral treatment | | | | | | |  |  |  |
| PLHIV: persons living with HIV; PWoH: persons without HIV; HDL-C: high density lipoprotein cholesterol | | | | | | | |  |  |

| **Appendix 5: Characteristics associated with high triglycerides** | | | | | | | | | |
| --- | --- | --- | --- | --- | --- | --- | --- | --- | --- |
| **Characteristic** | **N** | **Univariable** | | | | **Multivariable** | | | |
|  |  | **OR** | **95% CI** | | **P-value** | **OR** | **95% CI** | | **P-value** |
| **PLHIV vs PWoH** | 669 | 1.60 | 1.06 | 2.40 | 0.025 | 1.20 | 0.77 | 1.88 | 0.419 |
| **Age** | 669 | 1.05 | 1.03 | 1.07 | < 0.001 | 1.03 | 1.01 | 1.05 | 0.002 |
| **Female vs Male** | 669 | 0.71 | 0.49 | 1.03 | 0.068 | 0.76 | 0.47 | 1.23 | 0.264 |
| **Low HDL-C** | 669 | 2.00 | 1.37 | 2.91 | < 0.001 | 2.66 | 1.76 | 4.02 | < 0.001 |
| **Abdominal Obesity** | 669 | 1.82 | 1.26 | 2.64 | 0.002 | 1.73 | 1.11 | 2.71 | 0.016 |
| **High Blood Pressure** | 669 | 2.37 | 1.58 | 3.57 | < 0.001 | 1.67 | 1.05 | 2.64 | 0.029 |
| **Smoking (Past or Current)** | 669 | 2.62 | 1.37 | 5.00 | 0.004 | 2.07 | 0.966 | 4.45 | 0.062 |
| **Family History of Diabetes** | 669 | 1.75 | 1.14 | 2.69 | 0.011 | 1.42 | 0.89 | 2.28 | 0.141 |
| **Hemoglobin level** | 669 | 1.10 | 1.01 | 1.19 | 0.024 | 1.09 | 0.99 | 1.19 | 0.069 |
| **Alcohol Use (Past or Current)** | 669 | 1.65 | 1.01 | 2.70 | 0.047 | 0.89 | 0.49 | 1.63 | 0.705 |
| **Fruit Frequency** | 669 | 1.09 | 1.00 | 1.18 | 0.062 | 1.09 | 0.99 | 1.20 | 0.069 |
|  |  |  |  |  |  |  |  |  |  |
| **ART Duration** | 439 | 1.05 | 1.00 | 1.10 | 0.066 | 1.00 | 0.95 | 1.06 | 0.942 |
| **HIV RNA Viral Load** | 430 | 1.54 | 1.12 | 2.11 | 0.007 | 1.51 | 1.06 | 2.16 | 0.024 |
| **ART Regimen PI vs non-PI** | 439 | 2.00 | 1.17 | 3.42 | 0.011 | 2.06 | 1.12 | 3.79 | 0.019 |
|  |  |  |  |  |  |  |  |  |  |
| N: number of participants; OR: odds ratio; CI: confidence interval; ART: antiretroviral treatment; PI: protease inhibitor | | | | | | | | | |
| PLHIV: persons living with HIV; PWoH: persons without HIV; HDL-C: high density lipoprotein cholesterol | | | | | | | |  |  |

| **Appendix 6: Characteristics associated with low HDL-C** | | | | | | | | | |
| --- | --- | --- | --- | --- | --- | --- | --- | --- | --- |
| **Characteristic** | **N** | **Univariable** | | | | **Multivariable** | | | |
|  |  | **OR** | **95% CI** | | **P-value** | **OR** | **95% CI** | | **P-value** |
| **PLHIV vs PWoH** | 669 | 1.47 | 1.07 | 2.03 | 0.019 | 1.41 | 1.01 | 1.97 | 0.046 |
| **Female vs Male** | 669 | 2.00 | 1.47 | 2.72 | < 0.001 | 2.59 | 1.79 | 3.75 | < 0.001 |
| **High Triglycerides** | 669 | 2.00 | 1.37 | 2.91 | < 0.001 | 2.46 | 1.65 | 3.68 | < 0.001 |
| **Abdominal Obesity** | 667 | 0.69 | 0.51 | 0.95 | 0.022 | 0.46 | 0.32 | 0.65 | < 0.001 |
| **Hemoglobin** | 669 | 0.90 | 0.83 | 0.98 | 0.018 | 0.97 | 0.90 | 1.04 | 0.365 |
|  |  |  |  |  |  |  |  |  |  |
| **WHO Stage >=2 vs 1** | 439 | 3.57 | 1.50 | 8.46 | 0.004 | 2.30 | 0.93 | 5.69 | 0.072 |
|  |  |  |  |  |  |  |  |  |  |
| N: number of participants; OR: odds ratio; CI: confidence interval; WHO: World Health Organization | | | | | | | | |  |
| PLHIV: persons living with HIV; PWoH: persons without HIV; | | | | |  |  |  |  |  |

| **Appendix 7: Characteristics associated with abdominal obesity** | | | | | | | | | |
| --- | --- | --- | --- | --- | --- | --- | --- | --- | --- |
| **Characteristic** | **N** | **Univariable** | | | | **Multivariable** | | | |
|  |  | **OR** | **95% CI** | | **P-value** | **OR** | **95% CI** | | **P-value** |
| **Age** | 669 | 1.03 | 1.02 | 1.05 | < 0.001 | 1.04 | 1.02 | 1.06 | < 0.001 |
| **Female vs Male** | 669 | 3.52 | 2.53 | 4.91 | < 0.001 | 8.01 | 5.08 | 12.63 | < 0.001 |
| **Education Primary vs Tertiary** | 434 | 1.63 | 1.07 | 2.46 | 0.022 | 0.64 | 0.37 | 1.10 | 0.142 |
| **Education Secondary vs Tertiary** | 531 | 1.39 | 0.98 | 1.99 | 0.069 | 0.85 | 0.55 | 1.30 | 0.779 |
| **Low HDL-C** | 669 | 0.69 | 0.51 | 0.95 | 0.022 | 0.48 | 0.33 | 0.70 | < 0.001 |
| **High Triglycerides** | 667 | 1.82 | 1.26 | 2.64 | 0.002 | 1.78 | 1.13 | 2.82 | 0.013 |
| **High Blood Pressure** | 669 | 3.43 | 2.34 | 5.02 | < 0.001 | 2.33 | 1.46 | 3.72 | < 0.001 |
| **Family History of Hypertension** | 669 | 2.01 | 1.43 | 2.83 | < 0.001 | 1.72 | 1.13 | 2.61 | 0.011 |
| **Family History of Diabetes** | 669 | 1.98 | 1.35 | 2.91 | < 0.001 | 1.68 | 1.03 | 2.74 | 0.036 |
| **Income >=30,000 Naira vs Less** | 668 | 1.55 | 1.13 | 2.12 | 0.006 | 1.62 | 1.05 | 2.50 | 0.029 |
| **Married** | 669 | 1.35 | 0.95 | 1.91 | 0.091 | 1.32 | 0.83 | 2.09 | 0.236 |
| **Fruit Frequency** | 669 | 1.12 | 1.03 | 1.21 | 0.008 | 1.12 | 1.01 | 1.23 | 0.026 |
| **Alcohol Use (Past or Current)** | 668 | 1.52 | 0.97 | 2.38 | 0.066 | 2.46 | 1.40 | 4.33 | 0.002 |
|  |  |  |  |  |  |  |  |  |  |
| **ART Duration** | 437 | 1.05 | 1.01 | 1.09 | 0.028 | 1.02 | 0.97 | 1.07 | 0.548 |
| **CD4 Count <350 cell count/μL** | 434 | 0.46 | 0.26 | 0.82 | 0.009 | 0.63 | 0.32 | 1.26 | 0.194 |
| **HIV RNA Viral Load** | 428 | 0.74 | 0.52 | 1.03 | 0.075 | 0.77 | 0.51 | 1.18 | 0.232 |
|  |  |  |  |  |  |  |  |  |  |
| N: number of participants; OR: odds ratio; CI: confidence interval; ART: antiretroviral treatment; | | | | | | |  |  |  |
| PLHIV: persons living with HIV; PWoH: persons without HIV; HDL-C: high density lipoprotein cholesterol | | | | | | | |  |  |
